# Prevalence and demographics of anxiety disorders: a snapshot from a community health centre in Pakistan

**DOI:** 10.1186/1744-859X-6-30

**Published:** 2007-11-13

**Authors:** Hassan Khan, Saira Kalia, Ahmed Itrat, Abdullah Khan, Mahwash Kamal, Muhammad A Khan, Roha Khalid, Salman Khalid, Sunniya Javed, Sanniya Javed, Affan Umer, Haider Naqvi

**Affiliations:** 1 Medical College, Aga Khan University, Karachi, Pakistan; 2 Department of Psychiatry, Aga Khan University, Karachi, Pakistan

## Abstract

**Background:**

The developing world is faced with a high burden of anxiety disorders. The exact prevalence of anxiety disorders in Pakistan is not known. There is a need to develop an evidence base to aid policy development on tackling anxiety and depressive disorders in the country. This is the first pilot study to address the prevalence of anxiety disorders and their association with sociodemographic factors in Pakistan.

**Methods:**

A cross-sectional study was conducted among people visiting Aga Khan University Hospital (AKUH), a tertiary care facility in Karachi, Pakistan. The point prevalence of anxiety amongst the sample population, which comprised of patients and their attendants, excluding all health care personnel, was assessed using the validated Urdu version of the Hospital Anxiety and Depression Scale (HADS). The questionnaire was administered to 423 people. Descriptive statistics were performed for mean scores and proportions.

**Results:**

The mean anxiety score of the population was 5.7 ± 3.86. About 28.3% had borderline or pathological anxiety. The factors found to be independently predicted with anxiety were, female sex (odds ratio (OR) = 2.14, 95% CI 1.36–3.36, p = 0.01); physical illness (OR = 1.67, 95% CI 1.06–2.64, p = 0.026); and psychiatric illness (OR = 1.176, 95% CI 1.0–3.1, p = 0.048). In the final multivariate model, female sex (adjusted odds ratio (AOR) = 2, 95% CI 1.28–3.22) and physical illness (AOR = 1.56, 95% CI 0.97–2.48) were found to be significant.

**Conclusion:**

Further studies via nationally representative surveys need to be undertaken to fully grasp the scope of this emerging public health issue in Pakistan.

## Background

Anxiety is a state of apprehension, uncertainty, and fear arising from the anticipation of a realistic or imagined threatening event, often impairing physical and psychological functioning. General anxiety disorder (GAD) is the most common anxiety disorder, with a lifetime prevalence of 5.1% in the US [1]. The entity of general anxiety was originally conceptualized by Freud, who coined the term "anxiety neurosis". This included four major clinical syndromes: general irritability, chronic apprehension, anxiety attacks and secondary phobic avoidance [2]. The definition of GAD has changed over time and Diagnostic and Statistical Manual of Mental Disorders, 4^th ^Edition(DSM-4) takes persistent worry over 6 months along with three of the following six symptoms to be present: restlessness, fatigability, difficulty concentrating, irritability, muscle tension and sleep disturbance [3].

Anxiety disorders are common in the general population around the world [4]. They constitute a substantial proportion of the global burden of disease, and are projected to form the second most common cause of disability by 2020 [5]. These disorders exert significant financial burden on the global economy [6]. The exact prevalence of anxiety disorders in Pakistan is not known. Several studies have measured the prevalence of anxiety and depression together, with figures varying from 7% to 50% in different urban centers [7,8]. Overall, the prevalence estimates are higher when compared with other developing countries, and are twice the figures reported from Uganda [9], Lesotho [10] and Zimbabwe [11].

Pakistan, with an estimated population of 152 million, is the sixth most populous country in the world. It is projected to rise to the fourth spot by 2050 [12]. The country is undergoing a demographic transition, along with growing insecurity, terrorism, economical problems, political uncertainty, unemployment and disruption of the social fabric. About 39% of the population survives below the poverty line [13]. Thus, the association of anxiety disorders with the social, psychological and biological factors cannot be ignored and needs to be evaluated, and therefore formed an important objective of our research.

There is a need to develop an evidence base to aid policy development on tackling anxiety and depressive disorders. In order to develop an effective strategy we need prevalence estimates of anxiety disorders. Anecdotally, the number of people presenting to the hospitals with anxiety disorders has increased. However, there are no robust studies to back this claim. The demographic transition in the form of increased migration from social to urban centers, increasing poverty and psychosocial risk factors neatly accounts for such an increased burden of disease, but requires further evaluation.

The primary objective of our study was to estimate the point prevalence of anxiety in the people visiting a tertiary care hospital using a validated, concise and feasible screening instrument. Several reviews [14] show that the Hospital Anxiety and Depression Rating Scale (HADS) [15] is widely used as a brief self-rating instrument for both dimensional and categorical aspects of anxiety in both epidemiology and specialist care. In these settings the psychometric properties of the HADS are excellent [16,17]. The second main objective of this pilot study was to find out the relationship of anxiety with the demographic and social profile of the study population.

## Methods

### Study design and sample

This cross-sectional study was conducted among people visiting Aga Khan University Hospital (AKUH), a public tertiary care facility, in Karachi, Pakistan. The sample was collected via convenience sampling from the outpatient family medicine clinics and community health centre. The intensive care unit (ICU), emergency room, inpatients wards, psychiatric and surgery clinics were not part of the sampling frame. All persons associated with health care including doctors, nursing staff and medical students were excluded from the sample. All patients who had experienced death of a close relative within the past 3 months were excluded to avoid false positives due to grief reaction.

The sample population was between 18–65 years of age. We required a sample size of 424 subjects to fulfill the objectives of our study at a 95% confidence level. This sample size was calculated assuming a 50% prevalence of anxiety disorder and 5% bound of error. The sample was then inflated by 10% to account for non-respondents and incomplete questionnaires.

The study was conducted in compliance with the "Ethical principles for medical research involving human subjects" section of the Helsinki Declaration. The study protocol was discussed among the students and facilitating faculty for possible ethical concerns. All possible measures were taken to ensure the confidentiality of all participants. The questionnaires were given to subjects after seeking their verbal consent. The participants were asked to return the complete and filled questionnaire within an hour. At the time of administration participants were asked if they could read the Urdu questionnaire, if they were unable to do so, the principal investigator served as an interviewer. However, by and large the questionnaires were self-administered as the majority could read Urdu. A total of 406 (96%) people returned the completed questionnaire and were included in the analysis

### Outcome variables

The point prevalence of anxiety amongst the sample population was assessed using the validated Urdu version of HADS [18]. Furthermore, several questions assessing sociodemographics were also administered (see Additional file 1 for questionnaire).

The use of the Urdu validated version of HADS, keeping in view the widespread applicability of this questionnaire, adds to the strength of the study. HADS has been extensively used in various settings and studies. A recent review of 747 studies concluded that HADS performed well not only in hospital practice (for which it was first designed), but also in primary care patients and the general population [14]. The HADS consists of seven items for anxiety (HADS-A). The items are scored on a four-point scale from zero (not present) to three (considerable). The item scores are added, giving subscale scores on the HADS-A from zero to 21. In this study, valid HADS-A scale scores were defined as having answered all seven items on the HADS-A. In HADS-A the anxiety items are concentrated on general anxiety, and five of the items are close to the diagnostic criteria of generalized anxiety disorder (GAD). The concurrent validity of the HADS-A compared to other questionnaires for anxiety is described as between 0.60 and 0.80 on the anxiety subscale [14]. It has been reported that using cut-off score of ≥ 8 on HADS-A, GAD was detected with a sensitivity of 0.89 and a specificity of 0.75 [19].

### Statistical analysis

Data was double entered and analyzed in Statistical Package for Social Sciences 14.0 (SPSS, Inc., Chicago, IL, USA). Descriptive statistics were performed for mean scores and proportions. Chi-square and t tests were employed to look for associations between anxiety score categories and sex, marital status, family income, employment status, physical illnesses and other sociodemographic identifiers. Results were recorded as frequencies, means ± standard deviations (SD), and p values. Univariate logistical regression model was used to estimate the odds ratio and their confidence intervals.

Univariate covariates with a p value of ≤ 0.25 were entered into the multivariable model. Multivariable regression using a stepwise technique was conducted to adjust for confounders and determine the factors independently associated with anxiety. For all purposes, a p value of < 0.05 will be considered as the criteria of significance.

## Results

Of 406 study participants 279 (69%) were male and 127 (31%) were female. The mean age of the study participants was 33.22 ± 11.34 years. The mean score on the anxiety scale of the population was 5.7 ± 3.86. Table 1 gives the demographic characteristics of the study population. Table 2 shows the proportion of people with anxiety. Of the total sample, 28.3% of the people had borderline or pathological anxiety. Table 3 shows the percentage of population in different groups with respect to gender, employment status, marital status, physical illness and income along with the associated odds ratios. The population in each category was compared with respect to the anxiety status, which was defined as normal (anxiety score ≤ 7) and abnormal (borderline/abnormal: anxiety score ≥ 8) in accordance with HADS classification.

**Table 1:** Demographic characteristics of the study population

**Variables**	**Frequency (%)**
Total respondents	407(100.0)
Age (years)	
< 40	308 (75.6)
> 40	88 (21.4)
Sex	
Male	279 (68.5)
Female	127 (31.2)
Marital Status	
Married	245 (60.2)
Unmarried	162 (39.8)
Education	
Illiterate	17 (4.2)
Matric (10 years of education)	46 (11.3)
Intermediate (12 years of education)	70 (17.2)
Graduate/postgraduate	264 (64.9)
Current employment status	
Employed	114 (28.0)
Unemployed	245 (60.1)
Family income	
<5000	64 (15.7)
5000–20000	149 (36.6)
20000–35000	52 (12.8)
>35000	70 (17.2)

**Table 2:** Distribution of sample population by HADS anxiety categories

**Anxiety score**	**Frequency (%)**
Normal (0–7)	292 (71.7)
Borderline (8–10)	66 (16.2)
Abnormal (> 10)	49 (12.0)
Total	407 (100.0)

**Table 3:** The association of anxiety with demographic and social factors

		**Abnormal anxiety (n**_1_**= 115)**	**Normal (n**_2_**= 292)**	χ^2 ^**p value**	**OR (95% CI)**	**AOR (95% CI)**
Sex	Male	65	214	0.01	1 2.14 (1.36–3.36)	1 2.0 (1.28–3.22)
	Female	50	77			
Marital status	Married	65	180	0.342	1 1.24 (0.80–1.91)	-
	Not married	50	112			
Physical illness	No	68	209	0.026	1 1.67 (1.06–2.64)	1 1.56 (0.97–2.48)
	Yes	44	81			
Job	Not employed	65	180	0.385	1 1.38 (0.86–2.24)	-
	Employed	38	76			
Income	<5000	24	40	0.221	1 0.65 (0.35–1.21) 0.50 (0.22–1.14) 0.49 (0.23–1.05)	-
	5000–20000	42	107			
	20000–35000	12	40			
	> 35000	16	54			
Age	≤ 40	84	224	0.675	1 1.12 (0.66–1.89)	-
	> 40	26	62			
Psychiatric disorder	None	88	251	0.048	1 1.176 (1.0–3.1)	-
	Present	24	39			
Parent(s)	Alive	98	251	0.844	1 1.06 (0.56–2.03)	-
	Both dead	15	36			
Education	Illiterate	8	9	0.063	1 0.72 (0.24–2.22) 0.55 (0.19–0.62) 0.35 (0.13–0.93)	-
	Matric	18	28			
	Intermediate	23	47			
	Graduate/postgraduate	62	202			

n_1_, Abnormal anxiety level (HADS score ≥ 8); n_2_, normal (HADS score ≤ 7); OR, odds ratio; AOR, adjusted odds ratio.

The prevalence of anxiety for females was 39.4% compared to 23.3% for males (p = 0.01). Females were twice as likely than males to be anxious. People who had a physical disorder had higher levels of anxiety compared to those without physical illness (p = 0.026). Although anxiety was not associated with death of parents, it was nevertheless significantly associated (p = 0.042) with having a single parent (widowed mother). Interestingly, no association was found between anxiety and marital status (p = 0.342). Similarly, no association between anxiety and family income, and anxiety and occupational status existed.

In the final multivariate model, only gender was found to be significant (p = 0.02), while physical illness was marginally significant (p = 0.06)

## Discussion

To our knowledge this is the first study from Pakistan that measures the prevalence estimate of anxiety using a validated instrument. In Pakistan, the mean overall prevalence of anxiety and depression based on community samples is 33.62%, with a point prevalence of 45.5 % in women and 21.7% in men [20]. In neighbouring India, patients visiting primary care centers have reported prevalence estimates ranging from 21% to 57% [4]. Thus, the point prevalence of 28.2% overall and 39.9 % for women reported by our study concurs with the regional and local data reports.

The sociodemographic factors reported to be associated with anxiety in population are middle age, low level of education, marital status (divorced, widowed or separated), and being a housewife [20]. We did not find any significant association with marital status (single/married), age, income, employment status or parents being alive or dead. Although having a widowed mother was seen to be associated with high levels of anxiety we cannot justify the reason for this finding. We can hypothesize that the financial burden of caring for a widowed mother maybe the source of this underlying anxiety. However, it is beyond the scope of this study to identify the reason for this association, as we do not know if the respondents were directly involved in care of a widowed mother. Neither can we comment on the fact whether these widowed mothers may still be working and sharing the financial load of the family, or whether the underlying grief may make them an added dependant person in the family. The local data reports that loss of a parent, sibling or a family member is not associated with anxiety [21].

The higher level of anxiety disorders reported in women can be due to the psychosocial risk factor profile present for anxiety and depression in our setting. Some of these factors are linked to a very early marriage, hostile in-laws, financial dependency on males, and lack of intimate and confiding relationship with spouse [20]. Even though the existing socioeconomic adversity predisposes people to anxiety and depressive disorders in Pakistan, supportive family and friends may protect against development of these disorders [20].

Physical illness is taken as an ominous event in which well-being is compromised, leading to anxiety state and subsequent anxiety disorders. Thus, there is a strong and unique association between anxiety disorders and physical disorders. Several studies have reported anxiety to be associated with long-term chronic illness [22,23]. One study reported that almost two-thirds of the patients with chronic rheumatological disorders, suffered from a concomitant mood disorder [23]. Women in Pakistan generally have higher rates of reported illness than men. The main health problems reported by women in local surveys included, mental tension leading to headache, white vaginal discharge and body pains associated with fatigue [24]. However, these physical symptoms and illnesses could very likely be psychosomatic, especially keeping in consideration the socioeconomic pressures faced by women in the society. Nevertheless such symptoms have been the cause of much anxiety and frequent health care visits.

In Pakistan the health budget is 2% of the GNP, and the mental health budget is about 0.4% of this [25]. A majority of frequent users of medical resources have symptoms of anxiety and/or depression. It has been found that patients with a single anxiety disorder are 56% more likely to be a frequent user of medical services compared with patients with no anxiety disorder, and patients with comorbid anxiety and other psychiatric disorders were more than three times more likely to be a frequent user [26]. In the present scenario of scarce resources along with limited health care spending, anxiety disorders exert a significant "backbreaking" burden on the already dilapidated health service structure.

The study has certain limitations. The results are based only on data from a single, private tertiary care hospital that does not serve as representative of the whole population of the country. The study was conducted in an urban centre where levels of anxiety are expected to be high owing to the poor sociodemographic profile. Although the community health care center from where the sample was taken is visited by people from rural areas as well, we still believe that the study setting does limit the findings in being representative of the whole country. Furthermore, people visiting a hospital setting may be suffering from physical ailments and thus be more prone to anxiety, leading to high levels of anxiety in our sample population. As the design is cross-sectional, observation is made only at a particular duration in time; therefore we cannot conclude that the observations are a constant factor in the studied population or a finding at only one point in time. We also did not include patients who could not converse in Urdu, and this further restricts the generalization of our findings.

Secondly, we used convenience sampling, where it is not possible to quantify the error in extrapolating results to the entire population. Nevertheless, the use of the validated HADS questionnaire in our study strengthens the reliability of our results.

Taking a prudent view, while keeping in mind the limitations of this pilot study, we observe a high level of prevalence of anxiety amongst the population. Anxiety disorders are associated with major economical burden and should be considered an emerging public health threat, especially in a low-income country such as Pakistan. The evidence from the Pakistani population relating to the knowledge and practices of people faced with anxiety disorders in this setup needs to be established to ascertain in detail how the people respond to the psychosocial factors linked to anxiety and their means of coping with this morbid disease. Meanwhile, in the scenario of high prevalence of anxiety, heath planners need to come up with an effective strategy to manage anxiety disorders effectively on a community scale.

## Competing interests

The author(s) declare that they have no competing interests.

## Authors' contributions

HK and HN conceived the idea for the study. HK, SK, AI, AK, MAK, MK, RK, SJ, SJ, SK, AU collected and entered the data. HK, SK, HN wrote the manuscript. HK, AI, AK analyzed the data. All authors reviewed the final manuscript.

## Supplementary Material

Additional file 1 questionnaire Click here for file
