# Supplementary material for: Prevalence and demographics of anxiety disorders: a snapshot from a community health centre in Pakistan
Source: Ann Gen Psychiatry. 2007 Nov 13;6:30. doi: 10.1186/1744-859X-6-30 (PMC2186310; doi:10.1186/1744-859X-6-30)
Supplement: Additional file 1 — questionnaire [file 1744-859X-6-30-S1.doc]

**Prevalence and demographics of anxiety disorders: a snapshot from Pakistan**

This questionnaire is designed to investigate the prevalence and demographics anxiety disorders. Please sign and fill in the following questionnaire if you wish to participate. A strict confidentiality will be maintained for all the information. We appreciate your cooperation.

**Signatures----------------------**

***Please circle only one response.***

Age: ____ Years

Sex: 1. Male 2. Female

Marital Status: 1. Single 2. Engaged 3. Married 4. Divorced 5. Widowed

Place of origin:_____________

Education: 1. Matric 2. Intermediate 3. Graduate 4. Post Graduate 5. Other________

Parents Marital Status: 1. Divorced 2. Separated 3. Other____________

Parents : 1. Both alive 2. Both dead 3. Mother dead 4. Father dead

Time **(in months)** elapsed since death of parent(s): _______________

Family Income: 1) < 10000 2) 10000–30000 3) 30000–50000 4) 50000–100000 5) > 100000

Purpose of visit: 1) Patient 2) Patient relative 3) Other:________________

Comorbidity: 1) Medical illness____________ 2) Psychiatric Illness__________

Medication:_______________

**1) I feel tense or 'wound up':**

a) Most of the time

b) A lot of the time

c) From time to time, occasionally

d) Not at all

**2) I get a sort of frightened feeling as if something awful**

**is about to happen:**

a) Very definitely and quite badly

b) Yes, but not too badly

c) A little, but it doesn't worry me

d) Not at all

**3) Worrying thoughts go through my mind:**

a) A great deal of the time

b) A lot of the time

c) From time to time, but not too often

d) Only occasionally

**4) I can sit at ease and feel relaxed:**

a) Definitely

b) Usually

c) Not Often

d) Not at all

**5) I get a sort of frightened feeling like 'butterflies' in the stomach:**

a) Not at all

b) Occasionally

c) Quite Often

d) Very Often

**6) I feel restless as I have to be on the move:**

a) Very much indeed

b) Quite a lot

c) Not very much

d) Not at all

**7) I get sudden feelings of panic:**

a) Very often indeed

b) Quite often

c) Not very often

d) Not at all

**THANK YOU**

---------------------------------------------------------------------------------------------------------
